# Interpretative phenomenological analysis of the collaboration among healthcare professionals in the nursing home setting

**DOI:** 10.1016/j.rcsop.2024.100424

**Published:** 2024-02-18

**Authors:** Robin Crunenberg, Camille Charles, Alice Lallemand, Laetitia Buret, Geneviève Philippe, Olivier Ethgen

**Affiliations:** a Faculty of Medicine, Department of Pharmacy, Center for Interdisciplinary Research on Medicines (CIRM), University of Liège, Liège, Belgium; b Faculty of Medicine, Department of General Medicine, University of Liège, Liège, Belgium; c Faculty of Medicine, Department of Public Health, Epidemiology and Health Economics, University of Liège, Belgium, Liège, Belgium

**Keywords:** Interprofessional collaboration, Theory of planned behavior, Nursing homes, Interpretative phenomenological analysis

## Abstract

**Background:**

The theory of planned behavior (TPB) postulates that behavioral performance is guided by the intention to perform that behavior, influenced by attitudes, subjective norms, and perceived behavioral control. This framework can be applied to studying interprofessional collaboration among healthcare professionals to enhance patient safety and public health within nursing homes.

**Objectives:**

This study aimed to explore the roles of physicians, pharmacists, and nurses in the interprofessional collaboration process while identifying facilitators and barriers to effective collaboration among healthcare professionals.

**Methods:**

A qualitative interpretative phenomenological analysis (IPA) was carried out. Individual semi-structured interviews were conducted with 19 healthcare professionals. Qualitative data were then integrated and analyzed through the lens of the TPB.

**Findings:**

The IPA revealed the ten following themes, considered as both facilitators and barriers to interprofessional collaboration among healthcare professionals in the nursing home setting: communication, roles and responsibilities, willingness and recognition of collaboration's importance, mutual knowledge, trust, confidence, support from decision-makers, protocols, and technology were considered as facilitators while distance was considered as a barrier.

**Conclusion:**

Enhancing pharmacist-physician collaboration and refining pharmacist-nurse collaboration were essential goals. Intention for collaboration was influenced by attitudes (such as communication and mutual understanding), subjective norms (including support from decision-makers), and perceived behavioral control (such as confidence and adherence to protocols and technology). Addressing these factors could improve collaboration, enhancing residents' quality of life and professionals' sense of achievement.

## Highlights

• Enhanced quality of care within nursing homes necessitates effective interprofessional collaboration.
• Exploration highlighted the need for pharmacist-physician collaboration and for refining pharmacist-nurse collaboration.
• There is a need for more clarity between pharmacists and physicians, with each attributing the lack of willingness to collaborate to the other.
• Recommendations proposed by practitioners focus on augmenting the pharmacist's role and enhancing communication among decision-makers.
• Optimizing collaboration can yield multiple benefits, encompassing improved medication management and quality of life for health professionals.

## 1 Introduction

Synergy and collaboration among healthcare professionals improve patient outcomes and elevate quality care standards.^1^ Therefore, synchronized work among physicians, nurses, and pharmacists is needed. Interprofessional collaboration is a partnership between several healthcare professionals. The common goal is to structure collective action centered on patients' needs.^2^ This collective action is possible thanks to information sharing between professionals, its analysis, and synthesis.^3^ In addition, interprofessional collaboration leads to improved quality of care for patients.^4^

Particularly within care institutions like nursing homes, where geriatric patients suffer from multimorbidity and are subjected to polypharmacy,^5^ professional collaboration among healthcare professionals is imperative.

The added value of the collaboration between pharmacists and other healthcare professionals was already observed in nursing homes through a qualitative study.^6^ The results showed that all healthcare professionals' collaborative analysis and review of older individuals' medicines and prescriptions positively impacted patient safety and drug expenditure (^7^^,^^6^). In addition, better knowledge about medicines and their adverse health outcomes was provided following mutual education among healthcare professionals.^8^

Interprofessional collaboration and practices are then crucial, particularly in a nursing home setting, but are sometimes not efficient. Previous qualitative analyses on interprofessional collaboration among physicians, pharmacists, and nurses in nursing homes have shed light on several aspects. These studies have frequently uncovered communication and coordination issues among healthcare professionals.^9^ They further suggested that communication gaps between healthcare professionals could lead to medication errors^10^ and discrepancies in treatment approaches due to insufficient interprofessional collaboration.^11^

Implementing simple interventions such as Situation-Background-Assessment-Recommendation/Request (SBAR) communication tools in nursing homes could already improve collaboration among healthcare professionals. The SBAR principle implies a structured framework for communication, ensuring a clear and systematic exchange of essential information among healthcare professionals.^12^

Therefore, it remains essential to highlight all the perceived obstacles to this interprofessional collaboration in a specific context and region to be able to act on it to improve the well-being and physical and mental health of both healthcare professionals and patients.

Similar qualitative research in Belgium could provide valuable insights into the dynamics and challenges within Belgian nursing homes by offering a comparative perspective, highlighting unique cultural or systemic factors influencing interprofessional collaboration in Belgian healthcare settings. Indeed, pharmaceutical practice in Belgium seems to currently face significant challenges in terms of communication with healthcare professionals in long-term care facilities. Interprofessional collaboration between pharmacists and long-term care establishments is often hindered by communication barriers.^13^ Concerning education, while pharmacists in Belgium have a solid educational foundation, specific training programs for interprofessional collaboration with long-term care facilities are still limited.^14^ The laws governing pharmaceutical practice in Belgium provide a framework, but the specificity of interactions with long-term care facilities is not always clearly defined. Legislative adjustments may be necessary to promote enhanced collaboration and clarify respective roles(“^15^). Regarding interprofessional collaboration models, Belgium has introduced some initiatives, but they have not yet been widely implemented.^16^

Given the challenges identified, the main objective of the present qualitative approach is to better understand the current collaboration practices among healthcare professionals within nursing homes in Belgium by:

- Exploring the empirical landscape to comprehend perceptions, the roles of diverse collaborators, and the potential configurations of their collaboration;
- Understanding the barriers and facilitators that influence existing collaboration;
- Proposing strategies to improve interprofessional collaboration in Belgium.

Interprofessional collaboration improves patient care, but challenges persist, especially in nursing homes. Studying, using a qualitative design, and interprofessional collaboration in Belgian nursing homes are crucial to enhance care by addressing communication gaps and proposing strategies to improve them.

## 2 Methods

The Consolidated Criteria for Reporting Qualitative Research (COREQ),^17^ consisting of a 32-item checklist tailored for interviews and focus groups, was employed as a reporting tool in this manuscript (**See supplementary Materials S1).**

### 2.1 Study design

To allow an interpretative approach to the interprofessional collaboration status in the healthcare sector, we collected oral discourse, further transcribed into verbatim, from the targeted public using qualitative methods. Thematic analysis allows the categorization of explicit content of qualitative data by systematically identifying themes, patterns, and codes that emerge from the content, providing a structured and organized overview of the qualitative material (i.e., inductive approach).^18^ The interpretative phenomenological analysis (IPA) further delved into the subjective experiences, emotions, and meanings.^19^ The method has shown its relevance in health research.^20^ This method aims to bring to light what a lived personal and subjective experience implies for the individual through in-depth reflective interpretation. The target is to obtain a good understanding, through interviews of a small homogenous sample, of the participants' rich, wealthy lived experiences.

A deductive approach was also applied, further using existing theory (i.e., the Theory of Planned Behavior (TPB) to interpret our data).

### 2.2 Data collection

Semi-structured interviews of nursing home physicians, nurses, and pharmacists were conducted to collect qualitative data. The semi-structured interview guide was developed for further interpretative phenomenological analysis within the Theory of Planned Behavior (TPB) framework. From that perspective, the conception of the interview guide followed these successive steps: defining research objectives linked to TPB components, developing interview themes aligned with TPB elements, and crafting open-ended questions exploring participants' attitudes and perceptions related to the studied behaviors. The interview guide was internally validated by three physicians, three nurses, and three pharmacists (**Supplementary Material S2,** initially in French but translated here in English). The method allows the interviewee to respond freely and the interviewer to maintain a structured framework for the interview.

The interviews were conducted by an experienced qualitative researcher with over a decade of experience in a nursing setting with a background as a pharmacist. The interviewer (C.S.; M.Sc., MPH) (female gender) aimed for an unbiased and non-judgmental approach to encourage open sharing of participants' perceptions. No prior relationships existed between the interviewer and participants. The focus remained on understanding diverse perspectives, ensuring a neutral stance throughout the interviews. Regular reflexivity sessions were held with a scientific staff of researchers from different experiences and backgrounds to ensure data integrity.

The four topics investigated were the perception of the collaboration between the different healthcare professionals with a specific emphasis on the pharmacist's role in the collaboration, the form of the collaboration, the barriers and facilitators to collaboration, and the prospects for improving collaboration.

For qualitative interviews involving pharmacists, physicians, and nurses, a used sampling technique was the ‘snowball sampling’ method. We contacted a few key healthcare professionals using the random identification of members of the professionals' association. The method involves asking them to recommend others who could provide relevant perspectives, and we further expanded the participant network with the idea of allowing for the diversification of qualified participants, enriching qualitative analyses with varied and in-depth viewpoints. Interviews were conducted face-to-face, by phone call, or by video call according to the participants' preferences and recorded with an audio recorder with the participant's informed consent and filed notes made.

The study was conducted in the French community of Belgium. The sample of healthcare professionals to be interviewed also had to meet the following inclusion criteria: to be a physician, nurse, or pharmacist, to practice his or her profession in collaboration in a nursing home setting, and to understand and speak the French language. Following guidelines of the phenomenological method and the qualitative research, the sample size targeted for each professional representative was set at a minimum of 3 participants by profession studied, ideally until sample saturation. In our study, we indeed aimed to achieve theoretical saturation, defined as the point where additional data collection does not yield new theoretical insights or themes. The saturation was reached after 3 interviews (by profession interviewed) due to the convergence of themes and patterns across the initial interviews. Theoretical saturation was determined through comparisons between new obtained data with current findings from the literature.

The interviews were introduced by a presentation of the research project and an explanation of the interview process. An explanation was also given regarding the processing of data and the strict confidentiality and anonymization of the information collected.

### 2.3 Data processing method

In this qualitative study, we employed a mixed qualitative approach that integrated elements of content analysis embedded within the framework of IPA.^19^ This methodology was adapted to comprehensively explore and interpret the subjective experiences of interviewees:

- The first step was familiarization and transcription: The qualitative data, comprising interview transcripts, were carefully transcribed, and the researchers familiarized themselves with the content through multiple readings to gain a nuanced understanding of interviewees' experiences.
- The second step was labels identification and meaning units: following the principles of IPA, initial labels or ‘codes’ were established by two data coders (C.S. and R.C.) to capture significant aspects of the participants' experiences. Care was taken to avoid prematurely indexing information to preserve the richness of the qualitative data.
- The third step was properties and phenomena identification: As a part of the IPA, researchers deeply identify labels, aiming to understand the underlying properties and phenomena represented by labels. This step aimed to uncover the essence and nature of the experiences expressed by participants.
- The fourth step was organizing properties into categories: the properties identified were organized to identify overarching categories, allowing for a structured analysis while maintaining the richness of the qualitative data.
- The fifth step was deriving themes: applying both content analysis and IPA, the identified categories were analyzed to derive broader themes that elucidate the essential qualitative information. This step allowed for a comprehensive understanding of the underlying patterns and meanings.
- The sixth step was integration within IPA: The themes derived through content analysis were integrated within the interpretative phenomenological framework, aligning the broader themes with the deeper phenomenological exploration. This integration aimed to provide a holistic interpretation of participants' subjective experiences while embedding the richness of content analysis within the interpretative depth of IPA.

Both qualitative methods allowed for a robust analysis that combined the systematic categorization of content analysis with the nuanced exploration of IPA, yielding a comprehensive understanding of the qualitative data collected in this study.

In the context of this interpretative phenomenological analysis, researchers initially analyzed qualitative data independently. This individual analysis allowed each researcher to immerse themselves in the data, forming initial impressions and interpretations. Subsequently, they collaboratively reviewed their analyses, comparing and contrasting their findings to identify common themes and discrepancies, aligning with.^21^

### 2.4 Data interpretation

For the qualitative data analysis and interpretation, we used Ajzen's theoretical model: theory of planned behavior (TPB).^22^^,^^23^ The TPB serves as a theoretical framework by suggesting that behavior is directly related to the intention to perform this behavior. An intention refers to a person's planned decision to perform that behavior. It is a key component influencing whether someone plans to engage in a particular action. Intentions are shaped by attitudes, subjective norms, and perceived behavioral control, according to the theory:

- Attitudes toward behavior: This concept pertains to an individual's favorable or unfavorable evaluation of a behavior. For instance, if healthcare professionals have a positive attitude toward interprofessional collaboration, it will improve the likelihood of success in joint projects.
- Subjective norms: This concept represents the perceived social pressures impacting an individual's decision to engage in a behavior. For instance, if a nurse observes negative perceptions about the role of a pharmacist among their peers, it might discourage collaboration between them.
- Perceived behavioral control: This concept refers to an individual's perception of the ease or difficulty in performing a behavior. For instance, if a pharmacist perceives difficulties communicating with physicians, it diminishes their perceived control over establishing an efficient and effective collaborative relationship.

To interpret qualitative data using the TPB framework enabled us to explore how the three concepts shaped decision-making and behaviors by uncovering the motivations, social influences, and perceived control associated with particular behaviors expressed by interviewees.

In practice, several steps have therefore been applied:

- The first step was study design: The relevance of TPB concepts (i.e., attitudes, subjective norms, perceived behavioral control) was identified in collaboration between healthcare professionals.
- The second step was data collection: Questions probing individuals' attitudes were asked to assess participant opinions, perceived social influences, and perceived barriers related to the behavior of collaboration between healthcare professionals.
- The third step was data analysis: quotes reflecting favorable/unfavorable attitudes, perceived social pressures and control perceptions were identified and categorized between the three TPB concepts.
- The fourth step was pattern exploration: specific patterns (and interactions between patterns) among attitudes, subjective norms, and perceived control were highlighted.
- The fifth step was interpretation: how attitudes, subjective norms, and perceived control influenced behaviors were explained by demonstrating their role in participants' decision-making processes.

### 2.5 Ethics statement

The study protocol and data collection received approval from the Ethics Review Committee at the University Hospital of Liège (CHU of Liège) under reference 2022/318. Prior to the interviews, all participants provided informed written consent. The procedures involving human participants adhered to the principles outlined in the 1964 Helsinki Declaration and its subsequent amendments. Additionally, the study complied with European regulations on personal data (i.e., the General Data Protection Regulation or GPRD), ensuring the participants' anonymity throughout the research process.

## 3 Results

### 3.1 Study sample

A total of 19 professionals have been interviewed: eight community pharmacists, six physicians (i.e., four coordinating medical physicians and two general practitioners), and five nurses (i.e., one nurse and four head nurses). We encountered a dropout of two participants: one physician and one nurse withdrew from the study due to time constraints associated with their workload.

The studied sample comprised 13 women and six men. Nine healthcare professionals worked in a nursing home in urban areas and 10 in rural areas. The capacity of the different nursing homes varied between 30 and 162 beds. Of the 19 interviews, eight were conducted face-to-face, six by video and phone call. The mean interview duration was 33 min, with a minimum of 14 min and a maximum of 69 min. Of the 19 interviews conducted, all were transcribed verbatim, and nine were included in the interpretative phenomenological analysis (i.e., a sample of 3 interviews for each of the three health professions was enough to ensure sample saturation).

### 3.2 Role of healthcare professionals in collaborating

#### 3.2.1 Role of physicians

The coordinating physician held a pivotal organizational and advisory position within the nursing home. Their responsibilities also encompassed active participation in institutional management meetings, providing essential medical guidance for critical decision-making processes. They favored coordination among the nursing and medical staff, centralizing work routines, and implementing therapeutic protocols for the residents. Additionally, they guided other healthcare professionals, initiated training programs, and mediated conflicts among professionals when necessary.

General practitioners (GPs) were responsible for examining complaints from nursing home residents, conducting diagnoses, and prescribing appropriate treatments. Direct communication channels between GPs and nurses seemed established to ensure efficient healthcare delivery for residents. Indirect communication with pharmacists regarding residents' prescriptions occurred through nurses who either collected or received medications, or via phone calls facilitated by pharmaceutical assistants.

#### 3.2.2 Role of nurses

Nurses played a central role in monitoring the clinical aspects of nursing home residents, collaborating closely with physicians. They managed residents' treatments and prescriptions in collaboration with pharmacists. Additionally, nurses were responsible for administering therapeutics to the residents within the nursing home.

#### 3.2.3 Role of pharmacists

Pharmacists within nursing home settings were responsible for dispensing medications or therapeutic interventions as prescribed by physicians, facilitated through nurses' management. Their role encompassed interaction with physicians, entailing the communication of treatment availability, potential substitution options, validation of prescribed therapies, dosage verification, awareness regarding drug-drug interaction, and advice regarding medication use. Pharmacists were also implicated in advising nurses on medication regimens. Moreover, they checked specific authorizations for medications prescribed to nursing home residents and managed the medication process through the nursing home environment.

### 3.3 Collaborative overlaps in the three roles

All interviews revealed that the three professions are committed to patient-centered care. This collective focus ensures a holistic approach to resident well-being. Regarding medication management, there was a report of collaborative efforts toward ensuring safe and effective medication use in accordance with respective roles. These results from interview data highlighted the need for the possible improvement in collaborative efforts embedded within the roles of physicians, pharmacists, and nurses.

### 3.4 Factors impacting the collaboration between healthcare professionals

The thematic analysis, embedded in the IPA, allowed us to identify ten themes that impacted interprofessional collaboration between pharmacists, physicians, and nurses: communication, role and responsibilities, willingness to collaborate, knowledge of other collaborators, trust, recognition and self-confidence, support, rules and protocol, technology, and distance. Depending on the empirical landscape, these themes were sometimes considered as facilitators and sometimes as barriers to collaboration.

#### 3.4.1 Theme 1: communication

The interviews highlighted that nurses frequently played the role of intermediary between physicians and pharmacists, which most interviewees felt negatively. Indeed, nurses perceived this role as a resource and time-consuming. Physicians and pharmacists suggested that it would be easier to communicate more directly but needed to borrow this indirect communication. Pharmacists expressed fear of disturbing the physicians in their clinical practice. Written communication for the logistical aspects and the solutions to minor problems were perceived positively by most of the participants because of its benefits in tracking the exchanges concerning residents. Direct communication by phone or face-to-face was suggested as the best manner to solve health issues. Several participants pointed out the importance of having a unique and centralized contact to merge the issues the different healthcare professionals highlighted to ensure better communication and collaboration.

#### 3.4.2 Theme 2: roles and responsibilities

Most healthcare professionals highlighted the need for everyone to fulfill their roles and responsibilities. Regarding interprofessional collaboration, one professional's efforts significantly impact others' work. A well-executed job has a positive influence and frequently streamlines colleagues' work. However, all interviewed professionals perceived that at least one of their peers could improve or perform their work more effectively.

Some physicians and nurses anticipated collaboration with pharmacists for prescription checks (i.e., dosage, drug-drug interaction, and polypharmacy) to improve understanding of medications and guarantee the safety of nursing home residents.

*GP number 2: “I think it should be the role [of the pharmacist] to draw attention and ask if we are sure that it is the right dose for the medication prescribed. [Wonder] whether there was an error by the hospital or the attending physician. I know that sometimes that does not happen. In my opinion, it should be an essential role, which sometimes is not performed or performed well. […] And the pharmacist, it is perhaps hard to say, but somehow, I perceive him only as a drug deliverer for the nursing home.”*

*Nurse number 4: “But you have to organize the monitoring and the checking system, and that does not always seem essential to pharmacists. We prefer to give the drugs [without] checking that what is in them is the right treatment because if not, it is time-consuming. They [the pharmacists] do it sometimes, but every time we double-check [at the nursing home], we find medication errors. So, it is worth checking.”*

When the collaboration was perceived as efficient, the usefulness of the pharmacist's role in patient safety was highlighted.

*GP number 3: “At the pharmacy, they are very vigilant, which has a reassuring role […]. [The pharmacist] also wants to make no mistakes from the patient's point of view, and so that proves his seriousness.”*

Pharmacists and nurses frequently reported that the lack of physicians within the nursing home significantly negatively impacted the work of other healthcare professionals.

*Pharmacist number 2: “There are always medical physicians who do not come often or not at all at the nursing home… so there, it is a big barrier for communication and collaboration… […] the GP comes once every six months [so] we have a big issue with obtaining the prescriptions in due time, now, and for the future management of prescribed medicines.”*

*Nurse number 3: “The physicians are overwhelmed too, so I do not know how to meet. They will never come to a meeting […]. They are already in such demand on the left and right that I would be surprised if we… [succeeded in having a meeting with them].”*

Nurses suggested that they are perceived to have to fulfill a role that is not theirs, which can negatively influence the quality of their work given the workload for their practical role.

*Nurse number 4: “Or the pharmacy asks me to call a GP for lack of prescriptions. I call the GP and get yelled at by him, telling me not to bother him. Nevertheless, it is the pharmacy that asks me.”*

*Theme 3: Willingness to collaborate and awareness of the importance of collaboration.*

All the participants suggested that the willingness to communicate between each healthcare professional significantly impacted interprofessional collaboration.

*Physician number 3: “So if all the health partners do not collaborate or in any case are not vigilant, there is a link in the chain that gets stuck […].”*

Some participants highlighted the importance of proactivity in collaboration. Some physicians and pharmacists expressed their perceived need for more willingness from care partners to collaborate.

*Physician number 2: “The pharmacist could take a more proactive approach. If there was this proactivity, saying “we are here, and if you need, you call. I would call more easily, I think.”*

*Pharmacist number 1: “[…] we wrote a letter to invite the GPs […] to do medication plan reviews: it was us, the pharmacists, who were going to do the treatment plan review, but we had to meet the medical doctor to discuss, have his feedback and see if it would not be better to change the treatment plan. However, no medical doctor answered the letter in the affirmative!”*

The need to share health knowledge and compare views of health care was often suggested by the three representatives of healthcare professionals. Physicians and nurses expressed, for instance, a great interest in pharmacists sharing their knowledge on the proper use of medicines.

*Physician number 2: “Perhaps advice also in connection with the physiological specificities of the older person. Remember that some drugs must change according to kidney function, often altered in older residents. Afterward, it is also our role as physicians, but it can be interesting to collaborate and have a cross-section of perspectives.”*

*Theme 4: Mutual knowledge of health collaborators.*

It emerged that geographic proximity facilitated mutual recognition of each profession (and its related work environment). This proximity contributed to having more familiar communication among healthcare professionals, ultimately improving their collaborative efforts and efficiency.

*Theme 5: Trust in other health collaborators.*

Numerous participants highlighted the significance of mutual trust as a key factor positively influencing interprofessional collaborations. They suggested that clinical and management meetings may play a pivotal role.

*Physician number 3: “And besides that, they [the pharmacists] also proposed […] [to] the nurses, to go and see on-site at the pharmacy how a robot is, how it works, and reassure them that there are indeed three checks: the treatment plan, the robot itself, the pharmacist who manually rechecks. It reassures the nurses that at night, we can no longer spend time doing the work that has already been done twice before at the pharmacy.”*

Some participants pointed out that transparent, clear, and open communication between healthcare professionals would help avoid mistrust and improve collaboration.

*Physician number 3: “[…] it is the total obscurantism. Nevertheless, it is always easier to be obscure when you are far away and have no contact. When you are a pharmacist here if the patient is not happy, his/her family is not happy, and therefore, you risk losing customers while the guy over there does not care.”*

*Nurse number 3: “We do not always have the information… a piece of clear information. We sometimes have the impression that the pharmacist wants to make money… a little profit, uh…”*

A physician also clearly expressed that, as a coordinating doctor of EHPADs, he could strengthen mutual trust between the different partners and thus improve collaboration.

*Theme 6: Recognition and self-confidence.*

Recognition and self-confidence were also key factors that could impact collaboration between healthcare professionals.

Two pharmacists perceived a prevalent sense of professional hierarchy and a lack of physician appreciation for their expertise. The negative perception of pharmacists' skills among physicians instigated uncertainty among pharmacists regarding their credibility within collaborations. While some pharmacists occasionally hesitated to directly engage with physicians, opting to relay information through nurses, others advocated assertive communication with physicians. Nevertheless, they perceived a different attitude among younger physicians.

*Physician number 3: “[…] everyone has to make concessions […] and adapt to each other, and so the pharmacy also has to listen to the needs of the head nurses and suggest things for better management […].”*

*Theme 7: Support by decision-makers and funders.*

Some participants highlighted the importance of support from all decision-makers: nursing home directors and managers, pharmacy managers, and the health authorities.

Participants noted the importance of nursing home directors being more aware of the value of collaboration for the quality of care. However, It was perceived that some pharmacies were chosen to work with nursing homes based only on economic considerations (such as the cost of the pharmacy service or the percentage of rebates granted), which could lead to a certain distrust.

*Physician number 3: “[…] there are public contracts that are made with bids, and we have to choose the pharmacist who in the end is not necessarily the most competent but who offers either the most service or the most discounts, criteria that are not especially scientific or medical.”*

Financial support for medical and pharmaceutical managers, for example, through creating new jobs, can improve collaboration.

Finally, health authorities also played a role in supporting projects to improve collaboration between healthcare professionals.

*Pharmacist number 1: “Here, the National Institute of Health Insurance is creating this opportunity to do medical-pharmaceutical collaboration; it is started, so I hope it continues.”*

*Theme 8: Rules and protocols.*

Some participants suggested the importance of following predefined healthcare management rules and protocols. These make it possible to determine the formal and practical roles of each collaborator and the precise health procedures to be followed to avoid communication issues.

Other participants noted that collaboration between healthcare professionals was necessary to develop protocols. In addition, they highlighted the positive impact of implementing protocols on care quality and patient safety.

*Nurse number 2: “[…] our institution […] has embarked on an accreditation process. So, we had to develop a series of procedures to comply with the criteria proposed in the accreditation reference framework for medicines. As a result, we had to collaborate with the pharmacy […].*

*Theme 9: Technology.*

The majority of participants highlighted the need for automated and individualized drug preparation. However, one out of the three pharmacists disagreed, whereas the remaining pharmacists had an optimistic viewpoint: this system could facilitate a comprehensive overview of the medication patterns.

*Pharmacist number 2: “We have an overview, and we can check new treatments and see if there are no interactions or duplications. This robot production system allows close surveillance and monitoring of drug treatments.”*

Physicians and nurses have nuanced opinions regarding this system. Some noted that it could ease the work of the night shift nurses, but their opinion remained rather negative: the physicians have a limited choice of medications because of the limited storage space; the unit-dose delivery system prevents having a supply of medications, and the delivery of medications is no longer a daily process, which is problematic in case of emergency.

However, all the physicians and nurses noted that digital prescriptions were time-saving compared to paper ones.

*Theme 10: Distance.*

The health professionals interviewed suggested that the pharmacy's proximity, availability, and responsiveness significantly impacted the perceived quality of pharmacy services and further collaboration.

However, proximity alone was not enough to guarantee quality pharmaceutical service. According to some participants, the most important was that the pharmacy was available and responsive.

### 3.5 Integration of the themes identified within the frame of the TPB

According to the TPB, we highlighted that the ten themes impacting healthcare professional collaboration between professionals could be linked to the three theoretical concepts of the TPB.

#### 3.5.1 First concept: attitudes toward behavior

Attitudes toward behavior are defined by an individual's favorable or unfavorable assessment of endorsing a particular behavior. In this study, we focused on determining the favorable or unfavorable perceptions among pharmacists, nurses, and physicians regarding implementing collaborative efforts among healthcare professionals in the nursing home setting. Several factors impacted the positive or negative perception of interprofessional collaboration, consequently shaping participants' intentions to engage in collaboration or not actively. Three key themes emerged in this research:

- Communication: Professionals perceived collaboration unfavorably when communication was unclear, inefficient, and lacked fluency, resulting in a lack of incentives to engage better in collaboration.
- Roles and Responsibilities: Clearly defined roles and responsibilities among collaborators significantly facilitated and positively impacted professionals' perceptions of collaboration.
- Willingness to collaborate and recognition of collaborators: The proactive engagement of collaborators significantly boosted healthcare professionals' willingness to collaborate, impacting their overall positive perception of collaboration.

Furthermore, additional factors played a crucial role in shaping professionals' perceptions:

- Familiarity: Familiarity with collaborators facilitated more direct and warm professional relations, positively impacting how professionals perceived collaboration.
- Trust among collaborators: Mutual trust among collaborators positively influenced professionals' appreciation of collaboration.

These factors highlighted the intricate dynamics influencing professionals' attitudes toward collaborative efforts.

#### 3.5.2 Second concept: subjective norms

Subjective norms represent the perceived social influences guiding an individual's decision to engage in a behavior. Our study examined the social pressures influencing pharmacists, physicians, and nurses regarding collaboration within a nursing home setting. One prominent theme revealed by our research pertains to subjective norms: the support provided by decision-makers, health authorities, and stakeholders.

Healthcare professionals were more inclined to collaborate when they perceived approval and support from their hierarchical superiors. Previous studies have also emphasized the pivotal role of decision-makers and stakeholders in enhancing interprofessional collaboration. This support from authorities was reported as generating social pressure felt by healthcare professionals.

The choices made by managers in outsourcing pharmaceutical services to larger pharmacies or pharmacy chains was highlighted. While these options are often cost-effective, they might compromise the quality of collaboration among physicians, nurses, and pharmacists within the nursing home environment. These pharmacies, typically situated remotely, often need to provide daily drug deliveries, resulting in a lack of responsiveness during emergencies.

#### 3.5.3 Third concept: perceived behavioral control

Perceived behavioral control refers to an individual's perception regarding the ease or difficulty of performing a specific behavior, such as collaboration among healthcare professionals in the nursing home setting. We identified six key themes that highlight how participants perceived their control over interprofessional collaboration:

- Communication: Effective collaboration appears more feasible when communication between professionals is clear and persuasive.
- Familiarity: Professionals find it easier to trust and engage actively with colleagues they know personally, enabling direct contact to allow further effective collaboration.
- Recognition and (self-)confidence: When professionals experienced doubts about their skills or lack of recognition, they perceived collaboration as challenging.
- Rules and protocols: Defined rules and protocols facilitated collaboration among healthcare professionals.
- Technology: The introduction of new technologies was seen by some professionals as a facilitator and by others as a barrier to an efficient collaboration process. The ease or difficulty of collaboration using these technologies depended on their effectiveness and the proficiency of professionals in utilizing them.
- Proximity: The proximity of a pharmacy was reported as improving responsiveness in emergencies, thereby allowing a perception among nursing home professionals that collaboration was feasible.

## 4 Discussion

Our qualitative study aimed to investigate the (perceived) roles of physicians, pharmacists, and nurses in the interprofessional collaboration process and further identify the facilitators and barriers to this collaboration. Through a methodology employing content analysis and IPA, we explored the multifaceted roles of healthcare professionals in the collaborative process. The results are further discussed in light of the TPB.

### 4.1 Roles in the collaboration

In a nursing home setting, a discrepancy between formally defined roles and their practical implementation among healthcare professionals emerged from the IPA due to hierarchical structures and staff shortages, impeding efficient collaboration among physicians, nurses, and pharmacists. Indeed, hierarchical structures establish formal roles but can hinder teamwork.^24^ Moreover, professional responsibilities can overlap, leading to tensions and confusion among healthcare professionals, particularly regarding medication management.^25^ Staff shortages can also increase workload, diminishing the time dedicated to communication and collaboration.^26^^,^^27^

In summary, the gap between written roles and practical implementation, compounded by workforce shortages, significantly impacts communication and collaboration among healthcare professionals in nursing homes.

### 4.2 First concept: attitudes

The participants emphasized the importance of establishing specific rules and protocols to facilitate effective collaboration. This suggested a perceived need for explicit guidelines to govern interactions and behaviors among healthcare professionals, which did not seem to be currently well implemented in their nursing home. The focus on exploring attitudes toward behavior indicates a recognition that explicit guidelines could contribute to a more harmonious and efficient collaborative environment among healthcare professionals in the nursing home.

Further studies observed that standardized protocols also minimized errors in the nursing home setting, improving patient safety^28^.

### 4.3 Second concept: subjective norms

Our findings confirm previous literature highlighting the essential role of hierarchical approval and support in facilitating collaboration among healthcare professionals.^29^ Participants indeed expressed motivation to collaborate when they perceived support from their superiors. Furthermore, our results also highlighted the implications of certain managerial decisions, such as opting for larger pharmacy chains to supply the nursing homes. The service is cost-effective but compromises the quality of collaboration due to logistical constraints. Indeed, these pharmacies are often relocated, do not deliver the drugs daily, and, therefore, cannot respond to emergencies and favor healthcare professionals' collaboration and quality of care.

### 4.4 Third concept: perceived behavioral control

Recognizing the barriers to recognition and self-confidence in healthcare professionals' collaboration aligns previous research,^30^ highlighting pharmacists' impaired confidence hindering their collaboration with physicians. However, a positive shift was noted among younger physicians who increasingly recognized pharmacists' specialized skills. Introducing interprofessional education also emerged as a key feature for enhancing healthcare professionals' collaboration. Our study aligns with previous research highlighting mutual education as an efficient tool to improve understanding and proficiency.^24^

### 4.5 Conclusion emerging from the analysis from a TPB perspective

Our findings show that physicians, pharmacists, and nurses reported favorable inclinations toward collaboration in nursing homes. However, their intentions to initiate these collaborations remained challenging for the three health professions. Factors such as subjective norms, for instance, the absence of clear legal regulations, pose challenges, and perceived behavioral control issues, such as physicians' limited awareness regarding pharmacists' expertise and not efficient interpersonal skills, could represents barriers to the collaborative process. These observations align with previous work, highlighting the same issues regarding the TPB.^31^

### 4.6 Identified pathways for improving collaboration between healthcare professionals

Participants suggested strategies to improve collaboration among healthcare professionals in the nursing home. They emphasized the need for improved acknowledgment of the pharmacist's role and better communication between physicians and pharmacists. Suggestions often centered on the pharmacist's improved integration into collaboration. Physicians and nurses proposed proactive measures for pharmacists, like initiating communication through newsletters, direct and physical contact with physicians, and systematically daring to address any perceived collaboration issues.

The pharmacist, recognized as a drug expert, was further seen as pivotal in playing an educational role within the nursing home setting. This involved organizing training sessions for the nursing staff on proper medication use, providing advice during dispensing, explaining specific effects of medicines in older patients, highlighting possible drug-drug interaction, and being careful regarding prescription errors.

Participants also highlighted the perceived responsibilities of physicians, requiring an increased presence and willingness to collaborate.

Other proposed improvements predominantly focused on communication as a fundamental factor for efficient collaboration by planning regular face-to-face meetings among the three parties to discuss health issues and improve collaboration.

The importance of partnering with local pharmacies to understand the empirical landscape and ensure high-quality pharmaceutical service was also reported. Improving and securing digital systems for electronic drug prescription transmission to pharmacists was seen as beneficial and reducing nurses' workload.

To address communication challenges during breaks and staff rotations, some participants suggested designating a primary contact professional for exchanges between pharmacies and nursing homes.

Lastly, interest was expressed in pursuing an accreditation process as an incentive to further improve interprofessional collaboration.

Given the perspectives highlighted by participants, the SBAR principle could play a crucial role in overcoming barriers to collaboration among healthcare professionals in nursing homes. By providing a standardized framework for communication, SBAR establishes a common language and clear sequence for information exchange, reducing potential misunderstandings and ensuring mutual understanding. Moreover, the systematic nature of SBAR promotes more efficient communication by identifying key elements for improving communication. This allows healthcare professionals in nursing homes to share information concisely and relevantly, minimizing the risk of errors or confusion.

In the Belgian context, interprofessional collaboration in nursing homes seemed to faces challenges rooted in formal role hierarchies and organizational constraints. The study identified a notable gap between formally defined roles and their practical implementation, largely due to hierarchical structures hindering effective teamwork. Additionally, supplying in large chain pharmacy is common and often preferred by healthcare facility managers. However, based on our research, the choice to partner with large pharmacy chains can create barriers to collaboration due to various factors, such as the size of organizational structures and associated bureaucratic processes. Regarding the impact on collaboration, previous studies have also suggested that large pharmacy chains may sometimes have more rigid organizational structures, which can lead to difficulties in interprofessional collaboration. To enhance collaboration in Belgium, targeted measures can be implemented. Establishing specific rules and protocols, as highlighted by participants, would clarify expectations and interactions. Standardized protocols, a proven method in the literature,^30^ could improve collaboration and care quality. Addressing subjective norms involves reinforcing hierarchical support by raising awareness among managers about the benefits of effective interprofessional collaboration. Managerial decisions, including pharmacy service providers, should consider their impact on collaboration quality. To overcome recognition and confidence barriers, interprofessional education initiatives are recommended. Increasing physicians' presence, better integrating pharmacists, and jointly improving communication through regular face-to-face meetings can foster more effective collaboration.

### 4.7 Limitations of the study

The involvement of a female gender pharmacist with ten years of experience conducting interviews in the study could introduce a potential bias (i.e., Lincoln and Guba's principles of trustworthiness and reflexivity.^21^ Her extensive background might offer nuanced insights into pharmacist interactions in healthcare settings. However, it could influence the research by inadvertently imposing personal biases or preconceptions regarding the profession and subsequently regarding the role of the two others in participant responses or data interpretation. To mitigate the potential bias, the researcher maintained reflective practices, regularly assessed personal biases, and performed peer debriefing sessions.

A potential social desirability bias could also arise due to the intricate nature of interprofessional collaboration, potentially prompting participants to offer socially acceptable information. Participants were then informed about the data anonymization guarantee, ensuring the confidentiality of recorded information and encouraging spontaneous and sincere responses.

Moreover, the predominant focus on pharmacists in the interview guide could engender concerns about whether the emphasis on pharmacist-related inquiries impacted the participants' responses. The interview guide underwent rigorous internal validation by healthcare representatives and experts to limit the potential bias.

## 5 Conclusion

The quality of care in nursing homes relies on effective interprofessional collaboration. Findings from our qualitative study in the nursing home setting in Belgium indicated a need to enhance pharmacist-physician collaboration and refine pharmacist-nurse interaction.

There was a mutual perception gap between pharmacists and physicians, each faulting the other for lacking collaboration. Pharmacists seek better acknowledgment of their expertise, while physicians desire more proactive engagement from pharmacists. Pharmacists and nurses generally perceived their collaboration positively, but communication issues persist.

Proposed improvements focused on empowering pharmacists in their role (i.e., drug experts) and enhancing communication among all parties. Suggestions include proactive engagement by pharmacists (notably using mutual education), optimized communication during professionals' meetings, and proximity of physical pharmacies to facilitate better collaboration.

Efficient collaboration promises improved medication management, enhancing residents' quality of life and improving healthcare professionals' satisfaction, ultimately benefiting public health.

## Funding sources

This research receives no specific grant from funding agencies in the public, commercial, or not-for-profit sectors.

## Disclosure

All the authors declare no conflict of interest.

## CRediT authorship contribution statement

**Robin Crunenberg:** Writing – review & editing, Writing – original draft. **Camille Charles:** Writing – original draft. **Alice Lallemand:** Validation, Conceptualization. **Laetitia Buret:** Supervision. **Geneviève Philippe:** Validation, Supervision. **Olivier Ethgen:** Writing – review & editing, Supervision.

## Declaration of competing interest

The authors declare that they have no known competing financial interests or personal relationships that could have appeared to influence the work reported in this paper.

## Appendix A Supplementary data

Supplementary material S1: COREQ (COsolidates criteria for REporting Qualitative research) Checklist. Image 1

Supplementary material S2: Interview guide; interprofessional collaboration between pharmacists, physicians and nurses working for nursing homes. Image 2
