# Supplementary material for: Interpretative phenomenological analysis of the collaboration among healthcare professionals in the nursing home setting
Source: Explor Res Clin Soc Pharm. 2024 Feb 18;13:100424. doi: 10.1016/j.rcsop.2024.100424 (PMC10955404; doi:10.1016/j.rcsop.2024.100424)
Supplement: Supplementary file 2 — Supplementary material S2: Interview guide; interprofessional collaboration between pharmacists, physicians and nurses working for nursing homes. [file mmc2.docx]

**Interview guide: interprofessional collaboration between pharmacists, physicians and nurses working for nursing homes**

- Introduction

Hello, my name is … and I am one of the investigators of this research project. Thank you for participating in this interview.

- Presentation of the research

The topic of our research concerns interprofessional collaboration between physicians, nurses and pharmacists who work in or for nursing homes (and care homes). This interview has three objectives. The first one is to investigate the interprofessional collaboration between the three parties. The second one is to understand the barriers and facilitators to this collaboration. And the last one is to find leads to improve this collaboration.

- Data processing and confidentiality

If you agree, this interview will be audio-recorded to facilitate the transcription of your answers and will be deleted as soon as it is completed. Everything you say during this interview will remain confidential and anonymous. To ensure this anonymity, a coding system with a code for each participant will be used. Before starting the interview, you can indicate your consent by signing this document.

- Conduct of the interview

I am going to ask you different questions classified in 7 themes. You can answer freely, there is no right or wrong answer. You can interrupt me if you don't understand a question. Do you have any questions before we start?

- Questions

1. Introduction:

- Can you tell me more about your status within the nursing (and care) home?

- In what environment is the facility located? Rural, semi-rural, urban?

- What is the capacity of the facility?

1. Interprofessional collaboration in general:

- What is your perception of current collaboration between physicians and pharmacists in their work related to nursing (and care) homes?

- What is your perception of the current collaboration between nurses and pharmacists in their work related to nursing (and care) homes?

*Follow-up questions*:

- What is your perception of the current collaboration between physicians and pharmacists in their work in connection with nursing (and care) homes?

- How do you feel about the current collaboration between nurses and pharmacists in their work related to nursing (and care) homes?

1. Role of the pharmacist in the collaboration:

*Question for physicians and nurses*:

- In your professional practice in the nursing (and care) home, in what areas does the pharmacist collaborate with physicians and/or nurses? What does he/she do concretely?

*Question for pharmacists*:

- In your professional practice, in what areas do you collaborate with physicians and/or nurses working in nursing (and care) homes? What do you do concretely?

1. Form:

- What is the form of the collaboration? In the form of phone calls, email exchanges, working meetings?

*Sub-questions*:

- What is the frequency of contact?

- Where are the working meetings held?

1. Barriers:

*Question for physicians and nurses*:

- In your professional practice in the nursing (and care) home, what are the challenges of working with pharmacists?

*Question for pharmacists*:

- In your professional practice within the nursing (and care) home, what are the difficulties in collaborating with physicians and nurses?

*Follow-up question*:

- What factors make collaboration difficult?

1. Facilitators:

*Question for physicians and nurses*:

- In your professional practice in the nursing (and care) home, what factors facilitate collaboration with pharmacists?

*Question for pharmacists*:

- In your professional practice within the nursing (and care) home, what factors facilitate collaboration with physicians and nurses?

1. Interests:

- How might optimal collaboration between physicians, nurses, and pharmacists benefit:

° patients?

° physicians/nurses/pharmacists?

° society?

1. Perspectives:

- What are some ways that collaboration between physicians, pharmacists and nurses could be improved?

- What roles could or should the pharmacist play in collaboration with physicians/nurses?

- Closing the interview

- Is there anything else you would like to add before we close this interview?

- So this question closes our interview. Thank you for participating and helping me in this research project. I wish you a good day/evening.
